# Analysis of Heme and Non-Heme Iron Intake and Iron Dietary Sources in Adolescent Menstruating Females in a National Polish Sample

**DOI:** 10.3390/nu11051049

**Published:** 2019-05-10

**Authors:** Dominika Skolmowska, Dominika Głąbska

**Affiliations:** Department of Dietetics, Faculty of Human Nutrition and Consumer Sciences, Warsaw University of Life Sciences (SGGW-WULS), 159c Nowoursynowska Street, 02-776 Warsaw, Poland; dominika_skolmowska@sggw.pl

**Keywords:** iron intake, heme iron, non-heme iron, animal products, plant products, sources of iron, risk of anemia, female adolescents

## Abstract

Iron intake and heme/non-heme iron proportions are crucial for iron deficiency anemia prevention. Women of childbearing age are indicated by World Health Organization as the primary target group, but maintaining iron balance is particularly challenging for adolescents. The aim of the presented study was to analyze heme and non-heme iron intake and dietary sources in adolescent menstruating females in a national Polish sample. The study was conducted in a representative sample of adolescents (aged 15–20) who were recruited from all regions of Poland based on secondary school sampling (random quota sampling), with 1385 female adolescents being included in the sample. The iron intake was assessed using the previously validated IRONIC-FFQ (IRON Intake Calculation–Food Frequency Questionnaire). The intakes of iron, heme iron, non-heme iron, and iron from food product groups were assessed and compared with those of male adolescents (*n* = 1025) who were recruited from the same schools, as well as between sub-groups stratified by age, body mass index, anemia history, following vegetarian diet, applying iron supplementation and school type. Compared with male individuals, females were characterized by a lower intake of all forms of iron. It was stated that non-heme iron intake was highest in younger ones, overweight ones, vegetarian ones, and comprehensive school students. Female adolescents with anemia history were characterized by similar iron intake as others. For the target group, there is inadequate nutritional education and a necessity to broaden the knowledge about specific sources of iron.

## 1. Introduction

Anemia has been recognized by the World Health Organization (WHO) as one of the most significant global health problems that affects both developing and developed countries with serious consequences for human health [1]. WHO estimates that about 24.8% of the world population is anemic, which corresponds to about 1.62 billion people worldwide [2]. The etiology of anemia is multifactorial and complex, and the major contributor to the burden of this disease is iron deficiency, which is responsible for approximately 50% of anemia cases [3].

The reduction of the frequency of anemia prevalence is essential particularly for women of childbearing age, as a 50% reduction of the frequency of the disease in them is set as one of the WHO’s global targets for the year 2025, because women of childbearing age and pre-school children are particularly at risk of iron deficiency [4]. Furthermore, achieving reduction of anemia will be beneficial both for mothers and their offspring as the results of a meta-analysis have indicated a strong association between maternal iron deficiency anemia and adverse neonatal outcomes [5].

For a group of female adolescents, maintaining positive iron balance is challenging because of the growth spurt and maturation accompanied by higher iron requirements compared with male adolescents [6,7]. Moreover, inadequate dietary iron intake may deteriorate iron status in female adolescents [8], and it is quite common as 25–39% of females aged 10–19 years are characterized by inadequate iron intake [7]. Furthermore, for adolescents, anemia results in a wide range of serious health consequences, which are not observed for adults such as impaired mental development and physical growth and reduced school performance and work capacity [9,10].

Considering the long-term consequences of iron deficiency and iron deficiency anemia, it is necessary to individually assess the iron intake of adolescent girls and to provide adequate iron intake [11]. However, iron is present in food products in two forms, as heme iron, which is found in meat and other animal products, and as non-heme iron, which is found in both plant and animal products [12], that differ in their chemical form, absorption processes, and uptake mechanisms [13]. Heme iron is highly bioavailable (25–30% of this form is absorbed), although it represents a minor part of dietary iron [14,15], while the absorption of non-heme iron is more variable (1–10% of this form is absorbed) [16].

The aim of the presented study was to analyze heme and non-heme iron intake and iron dietary sources in adolescent menstruating girls in a national Polish sample.

## 2. Materials and Methods

### 2.1. Ethical Statement

The study was carried out at the Department of Dietetics, Warsaw University of Life Sciences (WULS-SGGW). The study was conducted according to the guidelines laid down in the Declaration of Helsinki and all procedures involving human subjects received the approval of the Ethics Committee of the Faculty of Human Nutrition and Consumer Sciences of the Warsaw University of Life Sciences (No 24/2018) All of the participants, as well as their parents or legal guardians provided written informed consent to participate in the study.

### 2.2. Study Group

The age of menarche in developed countries is 12–13 [17], but WHO indicates that regularity of cycles is obtained until the age of 15 [18]; therefore, the study was planned to be conducted in females who were older than 15 years to assess females of childbearing age. Moreover, WHO defines adolescents as individuals until the age of 19 or under the age of 20 [19]; therefore, this study was conducted for a group of females who were aged 15–20 years. In Poland, this age group is composed of secondary school students, and the net enrolment rate (NER) for this level of education in Poland is 89.01% [20].

To obtain a representative national Polish sample of adolescents, the stratified sampling was used as part of the recruitment procedure (random quota sampling with quotas for: (1) regions of Poland, and (2) gender proportions).

The study group was recruited using a two-stage sampling procedure: main phase and subsidiary phase to obtain representative sample from all of Poland’s voivodeships (the detailed procedure is presented in Supplementary Material).

The inclusion criteria were as follows:

1) Caucasian,
2) Adolescent aged 15–20,
3) For females declared regular menstruating,
4) Student of the randomly selected secondary school,
5) Written informed consent of adolescent to participate in the study,
6) Written consent of parents or legal guardians for participation of their children (for minors).

The exclusion criteria were as follows:

1) Pregnancy or current breastfeeding in case of female adolescents,
2) Over-reporters (assessed based on calculated iron intake and concluded for respondents exceeding iron tolerable upper intake level—UL of 45 mg [21]).

**Figure 1:**
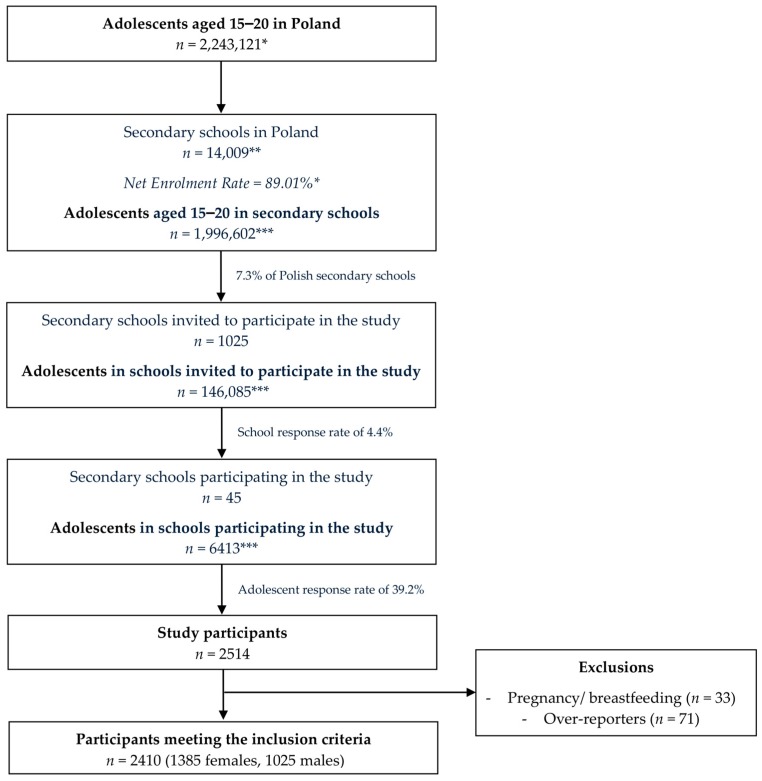
The detailed sampling procedure and recruitment of the studied group. * Central Statistical Office (CSO) data [20,22], ** Polish Ministry of National Education data [23], *** calculated based on Central Statistical Office (CSO) data [22].

The detailed sampling procedure and recruitment of the studied group is presented in Figure 1. The proportions of female and male respondents were verified in the regions of Poland (south-east, south-west, north-west, north, central, and west) compared with the Central Statistical Office (CSO) data [22]. The proportions were stated to not differ from the general Polish data (*p* = 0.3956; chi^2^ test). The analysis for male respondents is presented in Supplementary Material (Tables S1–S6).

### 2.3. Data Gathering

The information about intake of food products that are sources of iron was gathered using a previously validated IRON Intake Calculation–Food Frequency Questionnaire (IRONIC-FFQ) [24], and recalculated into iron intake using previously developed formulas [24] based on Polish food composition tables [25] for a Polish population. Iron intake is constant throughout the year [26], so the intake was assessed for a typical week from the previous year, and recalculated as per daily intake, according to the same approach as that used in a previous study [24].

Furthermore, the iron intake was calculated for specific food product groups: cereals, meat products, vegetables, nuts, fruits, cocoa products, eggs, potatoes, dairy products, fat, and fish products. Moreover, total animal products-derived iron and plant products-derived iron was also determined. Heme and non-heme iron intake was calculated based on a commonly applied assumption that heme iron is attributed to 40% of iron derived from animal products, while non-heme iron is attributed to 60% of iron derived from animal products and 100% of iron derived from plant products [27].

The total iron intake, heme iron intake, non-heme iron intake, and iron intake from the specific food product groups were compared between sub-groups stratified by gender, age, BMI, anemia history, following vegetarian diet, applying iron supplementation and school type.

Moreover, the total iron intake was compared with the recommended dietary allowance (RDA) level set by the Institute of Medicine [21]. The share of respondents, characterized by a recommended intake and intake lower than recommended, was compared between the sub-groups stratified by the gender, age, BMI, anemia history, following vegetarian diet, applying iron supplementation and school type. The analysis for male respondents is presented in Supplementary Material (Table S7).

### 2.4. Statistical Analysis

The respondents were stratified after taking into account the general characteristics to characterize the assessed female respondents compared with the male ones and in sub-groups, as follows:

- Gender—female respondents (*n* = 1385) and male respondents (*n* = 1025);
- Age—minor female respondents—aged <18 years (*n* = 1017) and adult female respondents—aged > 18 years (*n* = 368);
- BMI—assessed based on self-reported weight and height using the Quetelet equation (weight (kg)/height^2^ (m^2^))—underweight female respondents (*n* = 196), proper body mass female respondents (*n* = 1004), and excessive body mass female respondents (*n* = 185). However, BMI for minors was assessed using OLAF software [28] based on Polish growth reference curves that are specific for gender and age [29], whereas BMI was assessed for adults based on WHO classification [30];
- Anemia history—assessed based on self-reported declaration concerning diagnosed anemia over past year—female respondents declaring anemia diagnosed by a physician in the past year, interpreted as anemic ones (*n* = 229) and non-anemic ones (*n* = 1156);
- Following vegetarian diet—assessed based on self-reported declaration—non-vegetarian female respondents (*n* = 1300) and vegetarian female respondents (*n* = 85);
- Applying iron supplementation—assessed based on self-reported declaration concerning applying oral iron medication, multivitamin supplement or any other supplement containing iron—female respondents not applying iron supplementation (*n* = 1195) and female respondents applying iron supplementation (*n* = 190);
- school type—comprehensive secondary school female respondents (*n* = 492) and technical secondary school female respondents (*n* = 893).

The statistical analysis was conducted using Shapiro–Wilk test to verify normality of distribution, as well as chi^2^ test, Mann–Whitney U test and Kruskal–Wallis analysis of variance (ANOVA) to compare groups (due to a nonparametric distributions). It was performed using Statistica, version 8.0 (Statsoft Inc., Tulsa, OK, USA) and Statgraphics Plus for Windows 4.0 (Statgraphics Technologies Inc., The Plains, VA, USA), while *p* ≤ 0.05 was chosen as a level of significance.

## 3. Results

The baseline characteristics of the studied group is presented in Table 1.

The comparison of intake of various forms of iron along with that of iron intake from various sources in the national sample of Polish adolescents in the sub-groups of female and male respondents is presented in Table 2. It was stated that female respondents were characterized both by a lower total iron intake (*p* < 0.001) than the male respondents and by a lower heme iron, non-heme iron, animal iron, and plant iron intake (*p* < 0.001). Furthermore, their intakes of iron from cereals, meat products, cocoa products, eggs, potatoes, dairy products, and fish products were lower than that for male respondents, but their intake of iron from fruit was higher (*p* = 0.012).

The comparison of intake of various forms of iron along with that of iron intake from various sources in the national sample of Polish adolescents in the sub-groups of minor and adult female respondents is presented in Table 3. It was stated that minor female respondents were characterized not only by the higher total iron intake (*p* = 0.017), than the adult female respondents, but also by the higher non-heme iron (*p* = 0.011) and plant iron intake (*p* = 0.014). Furthermore, their intakes of iron from cereals and nuts were higher than for adult female respondents. Similar analysis for male adolescents (Supplementary Material—Table S1) revealed that minor male respondents were characterized not only by the higher heme iron and animal iron intake, than the adult male respondents, but also by the higher iron intake from meat products (*p* = 0.002) and cocoa products (*p* = 0.001). 

The comparison of intake of various forms of iron along with that of iron intake from various sources in the national sample of Polish female adolescents, in the sub-groups of underweight, proper body mass, and overweight female respondents is presented in Table 4. There were no differences observed in intake of various forms of iron between groups of underweight, proper body mass, and overweight female respondents. Furthermore, intakes of iron from cereals and cocoa products of proper body mass and overweight female respondents were higher than that for underweight female respondents. Similar analysis for male adolescents (Supplementary Material—Table S2) revealed that proper body mass male respondents were characterized by higher iron intake from cocoa products than the overweight male respondents (*p* = 0.020). Furthermore, proper body mass male adolescents had higher iron intake from eggs and fish products than underweight male adolescents. 

The comparison of intake of various forms of iron along with that of iron intake from various sources in the national sample of Polish female adolescents, in the sub-groups of anemic history and non-anemic history female respondents, is presented in Table 5. There were no differences observed in iron intake between groups of non-anemic and anemic history female respondents. Similar analysis for male adolescents (Supplementary Material—Table S3) revealed no differences in iron intake for the corresponding sub-groups. 

The comparison of intake of various forms of iron along with that of iron intake from various sources in the national sample of Polish female adolescents, in the sub-groups of vegetarian and non-vegetarian female respondents, is presented in Table 6. It was stated that non-vegetarian female respondents were characterized not only by the higher heme iron intake (*p* < 0.001), than the vegetarian female respondents, but also by the higher animal iron (*p* < 0.001). Vegetarian female respondents were characterized by a higher non-heme iron (*p* = 0.007) and plant iron intake (*p* < 0.001), than the non-vegetarian female respondents. Furthermore, their intakes of iron from cereals, vegetables, nuts, and fruit were higher than that for non-vegetarian female respondents, but their intakes of iron from meat products, potatoes, and fish products were lower. Similar analysis for male adolescents (Supplementary Material—Table S4) revealed that non-vegetarian male respondents were characterized by the higher heme iron and animal iron intake (*p* = 0.001), than the vegetarian male respondents. Furthermore, their intakes of iron from meat products and potatoes were higher than that for vegetarian male respondents.

The comparison of intake of various forms of iron along with that of iron intake from various sources in the national sample of Polish female adolescents, in the sub-groups of female respondents not applying and applying iron supplementation, is presented in Table 7. It was stated that female respondents applying iron supplementation were characterized by a higher total iron intake (*p* = 0.006) than the female respondents not applying iron supplementation and by a higher non-heme iron and plant iron intake. Furthermore, their intakes of iron from cereals, fruit, cocoa products, eggs, dairy products, and fish products were higher than that for female respondents not applying iron supplementation. Similar analysis for male adolescents (Supplementary Material—Table S5) revealed that intakes of iron from cereals, meat products, vegetables, nuts, fruit, eggs, and dairy products were also higher in a group of male respondents applying iron supplementation than that for male respondents not applying iron supplementation.

The comparison of intake of various forms of iron along with that of iron intake from various sources in the national sample of Polish female adolescents, in the sub-groups of female respondents from comprehensive and technical schools, is presented in Table 8. It was stated that female respondents from comprehensive schools were characterized both by a higher total iron intake (*p* = 0.001), than the female respondents from technical schools, and by a higher heme iron, non-heme iron, animal iron, and plant iron intake. Furthermore, their intakes of iron from cereals, fruit, eggs, potatoes, dairy products, and fats were higher than that for female respondents from technical schools. Similar analysis for male adolescents (Supplementary Material—Table S6) revealed no differences in intake of various forms of iron and intake of iron from various sources between groups of male respondents from comprehensive and technical schools.

**Figure 2:**
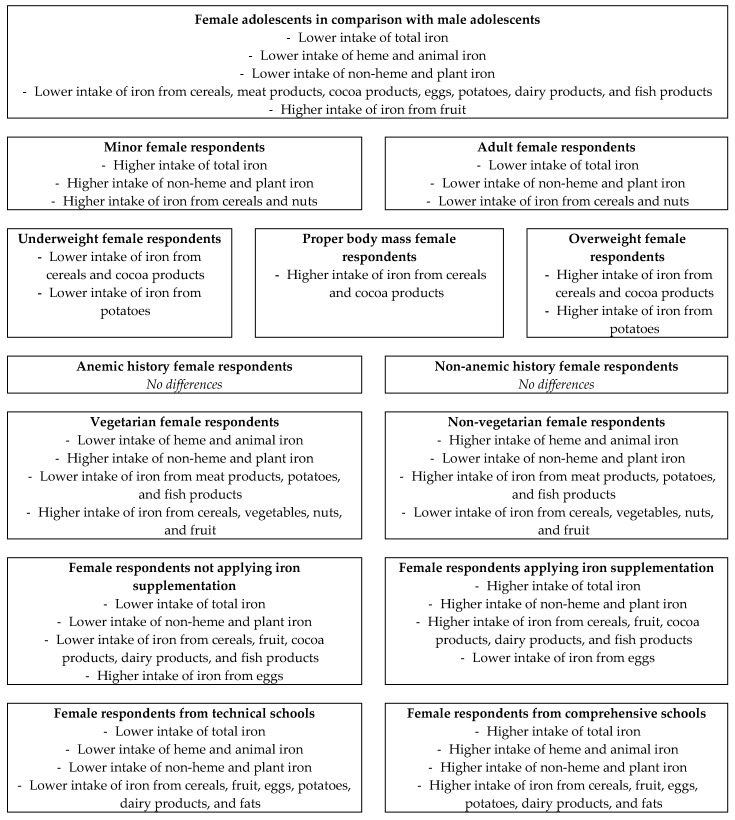
Summary of the presented results.

Summary of the obtained results is presented in Figure 2.

The share of individuals characterized by adequate or inadequate iron intake is presented in Table 9. The majority of female respondents were stated to be characterized by inadequate intake of iron (lower than the RDA) and they were more likely than male respondents to have iron intake below RDA level (*p* < 0.001). Adult female respondents were also more likely to be characterized by inadequate intake of iron, comparing with minor female respondents (*p* < 0.001).

## 4. Discussion

Based on the study carried out for a representative national Polish sample of adolescents, adolescent females were characterized by a significantly lower intake of all forms of iron (total iron, heme iron, non-heme iron, animal iron, and plant iron) compared to male adolescents. Furthermore, their intakes of iron from meat products, cocoa products, eggs, potatoes, dairy products, and fish products were lower than those for male respondents. These results agree with those of another study [31]. It is especially troublesome, as female individuals are commonly indicated as having too low iron intakes, that in Poland is stated both for not pregnant young women [32] and for pregnant ones [33], while their iron requirement is actually much higher than for male individuals [21].

Women and adolescent females often have both lower iron intake and lower total dietary intake of food products because of their dietary restraint [34], which directly influences their nutritional status including iron status [35]. However, adolescent females are particularly vulnerable to iron deficiency because of the combination of insufficient dietary iron intake and high iron losses during menstruation [36]. Menstrual blood loss can significantly contribute to iron depletion as it can be difficult for women to provide sufficient iron intake to compensate menstrual iron losses [14]. This is relevant because all female adolescents included in the study were menstruating, which can additionally affect their iron status [37]. Furthermore, female adolescents and young women are more susceptible to practice dieting compared to male adolescents, which results in reduced energy intake and limits consumption of certain food products [38]. Dietary restraints may lead to reduced meat intake and consequently lower iron intake [36]. Moreover, adolescents are likely to have poor dietary habits, which includes high consumption of fast foods that typically have high energy content and low nutrient density, which was observed for Polish adolescents in a representative sample of girls and young women [39]. Therefore, it is crucial to have adequate iron intake particularly for this group [40,41].

To combat iron deficiency, WHO indicates three main strategies, which can be used alone or in combination: oral iron supplementation, iron fortification of food, and dietary modification or diversification of current diet [1]. Iron fortification of staple food products is generally recommended for developing countries, whereas it is rather uncommon in Poland as primarily cereal products and corn flakes are iron-fortified [42,43]. Iron supplementation may be addressed to high-risk groups and represents a first-choice treatment as it is effective and inexpensive when administered properly [44]. However, conventional treatment of iron deficiency with iron supplements may result in gastrointestinal side effects such as stomach pain, nausea, or constipation [45]. Therefore, it seems that dietary-based interventions are the most appropriate way to improve iron status in young women and can act as an alternative to conventional treatment [45,46].

In the national Polish sample of adolescents, the primary source of iron for female adolescents was cereals; however, the primary source of iron for male adolescents was meat products, which had a similar share in the total daily iron intake. Furthermore, other studies conducted in a group of adolescents revealed that the primary source of iron for both adolescent females and males are cereals [41]. In a typical omnivorous diet, meat products may contribute even 40% or more of total iron intake [14], while in this study, they constituted 25.9% of total iron intake of female adolescents. Non-heme iron absorption is strongly influenced by dietary factors, while the food components have minor effect on heme iron bioavailability [47]. Plant products, which are source of non-heme iron, contain a number of iron inhibitors such as phytic acid, calcium, and polyphenols [48]. Therefore, it is important to provide both the appropriate amount of iron and to consider its bioavailability [49]. Such an approach should be targeted for a group of menstruating adolescent females to ensure that a positive iron balance is maintained.

Studies suggest that diets containing a significant share of cereal products, which are characterized by low iron bioavailability, may cause iron deficiency anemia among children [50]. Populations consuming predominantly plant-based diets with limited amount of meat can be at a risk of iron deficiency anemia because of poor absorption of iron from these diets [51]. It is worrying that in this study, the iron intake from meat products in female adolescents was two-fold lower than that in male adolescents, as it is well known that meat factor, which is an unidentified compound present in muscle tissue, enhances non-heme iron absorption from legume-based and cereal-based meals [52]. Furthermore, meat products act as a source of well-absorbed heme iron; therefore, in the study group, adolescent females should consider increasing the intake of meat products because they provide an adequate intake of heme iron [53]. Consequently, a properly balanced diet should contain an appropriate amount of iron with high bioavailability to satisfy increased iron requirements because of maturation during adolescence [54].

Consequently, overweight female adolescents were not characterized by a higher total iron intake compared to proper body mass and underweight female adolescents. Generally, obese adolescents tend to practice unhealthy dietary habits [55]. An unbalanced diet with inadequate or excessive intake of calories and other nutrients may not meet requirements for micronutrients such as iron. Therefore, although overweight female adolescents consume more energy compared to proper body mass female adolescents, they do not provide an adequate iron intake [56]. Overweight female respondents also had a higher iron intake from cocoa products, comparing to underweight female respondents, which may be associated with higher intake of chocolate and other chocolate sweets. Furthermore, obesity may affect iron status in overweight and obese females as iron absorption is 33% lower than that for proper body mass females despite having a similar iron intake [57]. Obese adolescents are also less likely to be perceived by specialists as anemic because of their excessive body mass; this issue can be particularly confusing while diagnosing anemia in this group. Therefore, both underweight and overweight adolescents should be considered while screening for anemia.

It was also observed that female respondents applying iron supplementation had higher total iron, non-heme iron and plant iron intake than the female respondents not applying iron supplementation. Furthermore, their intakes of iron from cereals, fruit, cocoa products, eggs and dairy products were higher than that for female respondents not applying iron supplementation. It may be assumed that females applying iron supplementation had higher overall nutritional awareness and are more likely to follow healthy dietary patterns, as it is commonly stated [58]. Such attitude may contribute not only higher iron intake but also supplementing this nutrient at the same time.

Female respondents from comprehensive secondary schools were characterized by a higher total iron, heme iron, non-heme iron, animal iron and plant iron intake compared to female respondents from technical secondary schools. Consequently, their intakes of iron from cereals, eggs and potatoes were higher than those for female respondents from technical schools. It may be suggested that females from comprehensive secondary school are characterized to have a healthier dietary pattern, which includes consuming more cereals and fruit compared to female adolescents from technical schools because of their higher nutritional awareness. This may be confirmed using the results of other Polish study in which health concerns were indicated as an important predictor of dietary behavior in girls and young women [59]. On the other hand, female respondents from technical secondary schools had a higher intake of iron from meat products than female respondents from comprehensive secondary schools and the higher intake contributed to higher heme iron intake.

Minor respondents had higher total iron, non-heme iron, and plant iron intake compared to adult female respondents. Moreover, they also consumed more vegetables and nuts, which may suggest that they follow a prudent pattern because either they are more aware of nutritional knowledge compared to adult respondents or their parents possibly influence their diet. Such a strong influence of parents and their diet was observed for a national sample of Polish children as the fruit consumption behavior and preferences of their mothers were determining factors for them [60].

Non-vegetarian female respondents had higher heme iron and animal iron intake than the vegetarian female respondents. Furthermore, their intakes of iron from cereals, vegetables, nuts, and fruit were higher than that for non-vegetarian female respondents, but their intake of iron from meat products and potatoes was lower. Such obtained results were expected. However, it should be emphasized that vegetarians are at greater risk of anemia development, comparing with non-vegetarians [61]. Vegetarian diet is characterized by the absence or lower share of highly bioavailable heme iron and by the presence of certain compounds of the plant foods which inhibit non-heme iron absorption [62]. Therefore, it is advisable for vegetarians to increase vitamin C and other organic acids intake to improve the iron bioavailability from their meals [62].

No differences of iron intake were reported between anemic history and non-anemic history female respondents. Furthermore, the iron intake from various dietary sources in anemic history female adolescents did not differ from the intake in non-anemic history females. Moreover, the predominant majority of adolescent females with diagnosed anemia did not meet dietary recommendations for iron. This is very disturbing as anemic female adolescents are especially supposed to have sufficient iron intake, because untreated anemia may lead to serious health implications and affect the quality of life [63]. In this study, anemic history females were not characterized by higher intake of any of iron sources, which may suggest that they did not follow a proper balanced diet. Generally, most adolescents at this age are not aware of their increased nutritional needs for pubertal growth, which should also include increased iron intake after the onset of menstruation [8,54]. One of the most important reasons of nutritional problems is the lack of nutritional knowledge; therefore, female adolescents, particularly, should be educated to prevent iron deficiency anemia as nutritional education is the most permanent and fundamental strategy to achieve changes in eating habits [64].

It should be emphasized that dietary guidelines targeted at adolescents are not realized, as a predominant majority of respondents had iron intake below recommended level, including those with anemia history. It may be assumed that nutritional awareness of adolescents is generally low and, as a consequence, adolescent females and males do not have appropriate dietary habits to prevent anemia development [54]. Therefore, it is necessary to formulate proper dietary recommendations to reduce the risk of anemia in the high-risk groups, including female adolescents, women of childbearing age, and pregnant women [65]. Moreover, such dietary recommendations should also be applied to treat the present anemia. However, recommendations based on the intake of specific nutrients may be confusing for the target groups so the food-based guidelines are more applicable and feasible. The important conclusion of the conducted study is relatively low contribution of meat products to the total iron intake in the group of female adolescents. As a result, it is necessary to formulate specific food-based guidelines, including combining products which are sources of non-heme iron with other nutrients, which increase its bioavailability.

In spite of the fact that the presented study was conducted in a large representative sample of Polish adolescents and important results were indicated, the limitations of the study must be mentioned. The most significant limitation of the presented study is associated with the fact that the iron intake assessed for the studied sample was not energy-adjusted. Such adjustment would be especially important, as in the study of Vandevijvere et al. [31] it was stated that while iron was adjusted for energy intake, adolescent females had significantly higher intakes than males. In the presented own study iron intake in a sub-group of females was significantly lower than for males, so observation of its intake related to energy intake would enable deeper conclusions. The other limitation is associated with the fact that a number of variable factors were assessed on the basis of the own declaration of participant (e.g., anemia history, body mass, following vegetarian diet). Moreover, adolescent respondents could underestimate or overestimate their own height or body mass, since this information was obtained based on the self-reported declarations of the participants. It may result in some inaccuracies of the obtained results, but as the aim of the study was to assess the large sample from the whole country and representative for all the regions, we had to base on some self-reported factors. Personal contact with the respondents and their general practitioners or assessment of the iron status would be, within this study, impossible. In order to use a quick and feasible questionnaire in the group of adolescents, there were no precise and detailed questions which referred to the type of applied iron supplement or to the details of anemia diagnosis. Another limitation of the conducted study is lack of the possibility of conducting blood count analysis. Such analysis would surely be beneficial for the obtained results and the conclusion, but taking into consideration the fact that the study was carried out in a large representative Polish sample, it would be very challenging.

## 5. Conclusions

In a representative national sample of Polish adolescents, females were characterized by lower intake of all forms of iron compared to males. Non-vegetarian female respondents had higher heme iron intake compared to vegetarian female respondents. Non-heme iron intake was higher in minor female respondents, overweight female respondents, vegetarian female respondents, and students from comprehensive secondary schools. Female adolescents with an anemia history were characterized by similar iron intake as others. Moreover, there is a need for nutritional education and educational campaigns among anemic and non-anemic adolescents, as no educational campaign related to iron deficiency and specific sources of iron has been conducted in Poland. These campaigns should be targeted at broadening knowledge by identifying and promoting various sources of well-absorbed iron. Such a strategy will be beneficial for an individual’s health during adolescence and for their future adulthood.

## Figures and Tables

**Table 1:** The baseline characteristics of the studied group.

Variable		Female Respondents (*n* = 1385)	Male Respondents (*n* = 1025)
Age (%)	Minors (age 15–17)	1017 (73.4%)	722 (70.4%)
	Adults (age 18–20)	368 (26.6%)	303 (29.6%)
Body mass index (BMI) (%)	Underweight	196 (14.2%)	54 (5.3%)
	Proper body mass	1004 (72.5%)	708 (69.1%)
	Overweight	185 (13.3%)	263 (25.6%)
Anemia history (%)	Anemic history	229 (16.5%)	41 (4.0%)
	Non-anemic history	1156 (83.5%)	984 (96.0%)
Following vegetarian diet (%)	Non-vegetarians	1300 (93.9%)	999 (97.5%)
	Vegetarians	85 (6.1%)	26 (2.5%)
Applying iron supplementation (%)	Not applying iron supplementation	1195 (86.3%)	902 (88.0%)
	Applying iron supplementation	190 (13.7%)	123 (12.0%)
Type of school (%)	Comprehensive school	492 (35.5%)	234 (22.8%)
	Technical school	893 (64.5%)	791 (77.2%)

**Table 2:** Comparison of intake of various forms of iron along with that of iron intake from various sources in the national sample of Polish adolescents, in the sub-groups of female and male respondents.

Intake of Iron		Female Respondents (*n* = 1385)			Male Respondents (*n* = 1025)			*p*-Value **
		Intake (%)	Mean ± SD	Median (25th–75th)	Intake (%)	Mean ± SD	Median (25th–75th)	
Intake of various forms of iron	Total iron (mg)	100	12.78 ± 7.06	11.01 (7.88–15.66) *	100	17.66 ± 9.21	15.57 (10.97–22.55) *	<0.001
	Heme-iron (mg)	13.1	1.68 ± 1.53	1.17 (0.71–2.13) *	17.2	3.03 ± 2.17	2.44 (1.48–4.06) *	<0.001
	Non-heme iron (mg)	86.9	11.10 ± 6.05	9.67 (6.85–13.75) *	82.8	14.63 ± 7.65	12.78 (9.06–18.73) *	<0.001
	Animal iron (mg)	32.9	4.21 ± 3.83	2.92 (1.76–5.34) *	42.9	7.58 ± 5.41	6.10 (3.69–10.14) *	<0.001
	Plant iron (mg)	67.1	8.57 ± 5.05	7.47 (5.12–10.58) *	57.1	10.09 ± 6.07	8.73 (5.67–12.99) *	<0.001
Intake of iron from various sources	Cereals (mg)	27.9	3.57 ± 2.27	3.07 (2.04–4.55) *	25.5	4.51 ± 3.19	3.80 (2.25–5.99) *	<0.001
	Meat products (mg)	25.9	3.30 ± 5.67	2.03 (0.98–4.32) *	34.8	6.14 ± 7.51	4.75 (2.53–8.21) *	<0.001
	Vegetables (mg)	17.3	2.22 ± 2.05	1.60 (0.79–2.89) *	14.0	2.48 ± 2.39	1.89 (0.97–3.93) *	0.059
	Nuts (mg)	8.1	1.03 ± 1.40	0.72 (0.00–1.45) *	6.0	1.05 ± 1.55	0.72 (0.00–1.45) *	0.966
	Fruit (mg)	5.6	0.72 ± 0.65	0.56 (0.37–0.92) *	4.0	0.70 ± 0.69	0.46 (0.28–0.92) *	0.012
	Cocoa products (mg)	4.1	0.52 ± 0.56	0.36 (0.18–0.67) *	3.5	0.62 ± 0.69	0.42 (0.21–0.79) *	0.001
	Eggs (mg)	4.1	0.52 ± 0.50	0.47 (0.31–0.63) *	5.0	0.89 ± 0.93	0.63 (0.31–1.10) *	<0.001
	Potatoes (mg)	2.9	0.37 ± 0.36	0.29 (0.21–0.43) *	3.1	0.55 ± 0.61	0.36 (0.21–0.64) *	<0.001
	Dairy products (mg)	2.1	0.27 ± 0.19	0.24 (0.15–0.35) *	2.0	0.35 ± 0.28	0.28 (0.18–0.44) *	<0.001
	Fat (mg)	1.1	0.14 ± 0.13	0.11 (0.06–0.17) *	1.0	0.17 ± 0.22	0.11 (0.06–0.20) *	0.103
	Fish products (mg)	0.9	0.11 ± 0.16	0.06 (0.00–0.13) *	1.1	0.20 ± 0.27	0.13 (0.01–0.22) *	<0.001

* Nonparametric distribution (verified using Shapiro–Wilk test; *p* ≤ 0.05), ** compared using Mann–Whitney U test (due to nonparametric distribution).

**Table 3:** Comparison of intake of various forms of iron along with that of iron intake from various sources in the national sample of Polish adolescents, in the sub-groups of minor and adult female respondents.

Intake of Iron		Minor Female Respondents (*n* = 1017)			Adult Female Respondents (*n* = 368)			*p*-Value **
		Intake (%)	Mean ± SD	Median (25th–75th)	Intake (%)	Mean ± SD	Median (25th–75th)	
Intake of various forms of iron	Total iron (mg)	100	12.99 ± 7.08	11.19 (8.09–15.80) *	100	12.19 ± 6.99	10.31 (7.50–14.92) *	0.017
	Heme-iron (mg)	13.2	1.71 ± 1.57	1.17 (0.70–2.17) *	13.3	1.62 ± 1.41	1.16 (0.74–2.05) *	0.704
	Non-heme iron (mg)	86.8	11.28 ± 6.06	10.03 (7.05–13.91) *	86.7	10.57 ± 6.02	9.02 (6.45–13.02) *	0.011
	Animal iron (mg)	32.9	4.27 ± 3.93	2.93 (1.75–5.41) *	33.1	4.04 ± 3.53	2.90 (1.86–5.12) *	0.704
	Plant iron (mg)	67.1	8.72 ± 5.07	7.70 (5.29–10.74) *	66.9	8.15 ± 4.98	7.05 (4.62–10.12) *	0.014
Intake of iron from various sources	Cereals (mg)	27.9	3.62 ± 2.26	3.18 (2.13–4.57) *	28.1	3.43 ± 2.29	2.83 (1.85–4.36) *	0.024
	Meat products (mg)	25.9	3.37 ± 3.72	2.06 (0.97–4.38) *	25.6	3.12 ± 3.29	1.94 (1.02–3.93) *	0.739
	Vegetables (mg)	17.4	2.26 ± 2.02	1.60 (0.79–3.04) *	17.3	2.10 ± 2.12	1.44 (0.79–2.93) *	0.054
	Nuts (mg)	8.3	1.08 ± 1.48	0.72 (0.18–1.45) *	7.3	0.89 ± 1.14	0.54 (0.00–1.45) *	0.013
	Fruit (mg)	5.6	0.73 ± 0.62	0.56 (0.37–0.93) *	5.8	0.71 ± 0.71	0.55 (0.28–0.92) *	0.230
	Cocoa products (mg)	4.1	0.54 ± 0.58	0.36 (0.18–0.69) *	4.0	0.49 ± 0.51	0.36 (0.15–0.63) *	0.303
	Eggs (mg)	2.8	0.52 ± 0.48	0.47 (0.31–0.63) *	4.4	0.53 ± 0.54	0.45 (0.31–0.63) *	0.824
	Potatoes (mg)	2.8	0.36 ± 0.35	0.29 (0.21–0.43) *	3.2	0.39 ± 0.38	0.29 (0.21–0.43) *	0.145
	Dairy products (mg)	2.1	0.27 ± 0.19	0.24 (0.16–0.35) *	2.2	0.27 ± 0.21	0.24 (0.13–0.34) *	0.456
	Fat (mg)	2.1	0.13 ± 0.13	0.11 (0.06–0.17) *	1.1	0.14 ± 0.14	0.10 (0.06–0.17) *	0.461
	Fish products (mg)	1.0	0.11 ± 0.15	0.06 (0.00–0.13) *	1.0	0.11 ± 0.18	0.06 (0.00–0.13) *	0.510

* Nonparametric distribution (verified using Shapiro–Wilk test; *p* ≤ 0.05), ** compared using Mann–Whitney U test (due to nonparametric distribution).

**Table 4:** Comparison of the intake of various forms of iron along with that of iron intake from various sources in the national sample of Polish female adolescents, in the sub-groups of underweight, proper body mass, and overweight female respondents.

Intake of Iron		Underweight Female Respondents (*n* = 196)			Proper Body Mass Female Respondents (*n* = 1004)			Overweight Female Respondents (*n* = 185)			*p*-Value **
		Intake (%)	Mean ± SD	Median (25th–75th)	Intake (%)	Mean ± SD	Median (25th–75th)	Intake (%)	Mean ± SD	Median (25th–75th)	
Intake of various forms of iron	Total iron (mg)	100	12.68 ± 6.23	10.44 (7.82–14.75) *	100	12.81 ± 7.12	11.01 (7.82–15.63) *	100	13.72 ± 7.85	11.59 (8.20–17.15) *	0.113
	Heme-iron (mg)	13.3	1.69 ± 1.42	1.12 (0.77–2.06) *	13.3	1.70 ± 1.54	1.18 (0.70–2.15) *	12.2	1.68 ± 1.55	1.15 (0.69–2.02) *	0.905
	Non-heme iron (mg)	86.7	10.99 ± 5.20	9.14 (6.51–12.94) *	86.7	11.12 ± 6.09	9.64 (6.85–13.77) *	87.8	12.04 ± 6.97	10.23 (7.33–14.91) *	0.068
	Animal iron (mg)	33.3	4.22 ± 3.73	2.81 (1.92–5.14) *	33.1	4.24 ± 3.84	2.96 (1.75–5.38) *	30.6	4.20 ± 3.87	2.88 (1.73–5.04) *	0.905
	Plant iron (mg)	66.7	8.46 ± 4.20	7.12 (4.60–9.99) *	66.9	8.58 ± 5.06	7.47 (5.13–10.56) *	69.4	9.52 ± 6.18	8.08 (5.63–11.72) *	0.059
Intake of iron from various sources	Cereals (mg)	27.9	3.54 ± 1.81	2.77 (1.64–4.87) *^A^	28.2	3.61 ± 2.28	3.14 (2.08–4.56) *^B^	28.2	3.87 ± 2.75	3.11 (2.15–4.82) *^B^	0.016
	Meat products (mg)	26.1	3.31 ± 3.41	1.84 (1.08–3.93) *	26.1	3.34 ± 3.63	2.10 (0.98–4.35) *	24.2	3.32 ± 3.73	1.98 (0.92–4.22) *	0.787
	Vegetables (mg)	17.1	2.17 ± 1.90	1.76 (0.90–3.20) *	16.8	2.15 ± 1.96	1.60 (0.79–2.89) *	18.9	2.60 ± 2.60	1.76 (0.97–3.23) *	0.291
	Nuts (mg)	8.1	1.03 ± 1.09	0.54 (0.00–1.45) *	8.2	1.05 ± 1.46	0.72 (0.03–1.45) *	7.9	1.08 ± 1.36	0.72 (0.02–1.45) *	0.353
	Fruit (mg)	5.6	0.71 ± 0.50	0.56 (0.37–0.92) *	5.5	0.71 ± 0.61	0.55 (0.37–0.92) *	6.0	0.82 ± 0.93	0.56 (0.28–1.02) *	0.857
	Cocoa products (mg)	4.1	0.52 ± 0.47	0.28 (0.12–0.58) *^A^	4.2	0.54 ± 0.59	0.36 (0.18–0.69) *^B^	4.1	0.56 ± 0.55	0.42 (0.21–0.79) *^B^	0.005
	Eggs (mg)	4.2	0.53 ± 0.60	0.47 (0.31–0.63) *	4.1	0.52 ± 0.47	0.47 (0.31–0.63) *	3.6	0.50 ± 0.54	0.31 (0.31–0.63) *	0.748
	Potatoes (mg)	2.8	0.36 ± 0.30	0.29 (0.14–0.36) *^A^	2.9	0.37 ± 0.35	0.29 (0.21–0.43) *^AB^	3.2	0.44 ± 0.43	0.36 (0.21–0.50) *^B^	0.003
	Dairy products (mg)	2.1	0.27 ± 0.21	0.25 (0.15–0.38) *	2.1	0.27 ± 0.19	0.24 (0.15–0.34) *	2.0	0.28 ± 0.20	0.25 (0.13–0.63) *	0.607
	Fat (mg)	1.1	0.14 ± 0.14	0.11 (0.06–0.17) *	1.1	0.14 ± 0.14	0.11 (0.06–0.17) *	1.0	0.14 ± 0.13	0.11 (0.06–0.17) *	0.443
	Fish products (mg)	0.9	0.11 ± 0.17	0.06 (0.00–0.13) *	0.8	0.11 ± 0.16	0.06 (0.00–0.13) *	0.8	0.10 ± 0.14	0.06 (0.00–0.13) *	0.220

* Nonparametric distribution (verified using Shapiro–Wilk test; *p* ≤ 0.05), ** compared using Kruskal–Wallis analysis of variance (ANOVA) (due to nonparametric distribution), values with different letters (A, B) differ in rows.

**Table 5:** Comparison of the intake of various forms of iron along with that of iron intake from various sources in the national sample of Polish female adolescents, in the sub-groups of anemic history and non-anemic history female respondents.

Intake of Iron		Anemic History Female Respondents (*n* = 229)			Non-Anemic History Female Respondents (*n* = 1156)			*p*-Value **
		Intake (%)	Mean ± SD	Median (25th–75th)	Intake (%)	Mean ± SD	Median (25th–75th)	
Intake of various forms of iron	Total iron (mg)	100	13.32 ± 7.52	10.92 (8.05–16.34) *	100	12.70 ± 7.03	11.037.84–15.59) *	0.447
	Heme-iron (mg)	12.8	1.70 ± 1.74	1.13 (0.68–2.09) *	13.2	1.68 ± 1.49	1.19 (0.71–2.15) *	0.320
	Non-heme iron (mg)	87.2	11.62 ± 6.41	9.62 (7.09–14.47) *	86.8	11.02 ± 6.05	9.69 (6.77–13.60) *	0.291
	Animal iron (mg)	31.8	4.24 ± 4.35	2.83 (1.71–5.21) *	33.1	4.21 ± 3.72	2.98 (1.78–5.37) *	0.320
	Plant iron (mg)	68.2	9.08 ± 5.43	7.69 (5.44–11.15) *	69.9	8.50 ± 5.06	7.39 (5.04–10.43) *	0.188
Intake of iron from various sources	Cereals (mg)	28.3	3.77 ± 2.42	3.20 (2.21–4.54) *	27.9	3.54 ± 2.27	3.04 (1.98–4.56) *	0.152
	Meat products (mg)	24.7	3.30 ± 3.98	1.80 (0.91–4.15) *	26.0	3.31 ± 3.53	2.07 (0.99–4.34) *	0.278
	Vegetables (mg)	18.2	2.43 ± 2.33	1.76 (0.79–3.23) *	17.2	2.18 ± 2.01	1.60 (0.79–2.89) *	0.247
	Nuts (mg)	8.2	1.10 ± 1.50	0.55 (0.00–1.63) *	8.0	1.02 ± 1.38	0.72 (0.00–1.45) *	0.853
	Fruit (mg)	5.7	0.76 ± 0.81	0.55 (0.28–0.93) *	5.6	0.72 ± 0.61	0.56 (0.37–0.92) *	0.623
	Cocoa products (mg)	3.7	0.49 ± 0.43	0.35 (0.15–0.75) *	4.2	0.53 ± 0.59	0.36 (0.18–0.64) *	0.752
	Eggs (mg)	4.1	0.55 ± 0.54	0.47 (0.16–0.39) *	4.1	0.52 ± 0.49	0.47 (0.31–0.63) *	0.971
	Potatoes (mg)	3.0	0.40 ± 0.44	0.29 (0.21–0.43) *	2.9	0.36 ± 0.34	0.29 (0.21–0.43) *	0.660
	Dairy products (mg)	2.1	0.29 ± 0.19	0.26 (0.16–0.39) *	2.1	0.27 ± 0.20	0.24 (0.15–0.34) *	0.154
	Fat (mg)	1.1	0.14 ± 0.12	0.11 (0.06–0.17) *	1.1	0.14 ± 0.11	0.11 (0.06–0.17) *	0.420
	Fish products (mg)	0.9	0.11 ± 0.17	0.06 (0.00–0.13) *	0.9	0.11 ± 0.16	0.06 (0.00–0.13) *	0.459

* Nonparametric distribution (verified using Shapiro–Wilk test; *p* ≤ 0.05); ** compared using Mann–Whitney U test (due to nonparametric distribution).

**Table 6:** Comparison of the intake of various forms of iron along with that of iron intake from various sources in the national sample of Polish female adolescents, in the sub-groups of vegetarian and non-vegetarian female respondents.

Intake of Iron		Vegetarian Female Respondents (*n* = 85)			Non-Vegetarian Female Respondents (*n* = 1300)			*p*-Value **
		Intake (%)	Mean ± SD	Median (25th–75th)	Intake (%)	Mean ± SD	Median (25th–75th)	
Intake of various forms of iron	Total iron (mg)	100	13.96 ± 7.87	12.31 (8.10–18.76) *	100	12.70 ± 7.01	10.98 (7.85–15.60) *	0.186
	Heme-iron (mg)	5.1	0.71 ± 1.06	0.42 (0.25–0.70) *	13.8	1.75 ± 1.54	1.22 (0.76–2.19) *	<0.001
	Non-heme iron (mg)	94.9	13.25 ± 7.59	11.43 (7.40–17.38) *	86.2	10.96 ± 5.92	9.57 (6.80–13.47) *	0.007
	Animal iron (mg)	12.7	1.77 ± 2.64	1.06 (0.63–1.76) *	34.4	4.37 ± 3.84	3.06 (1.89–5.47) *	<0.001
	Plant iron (mg)	87.3	12.19 ± 7.43	10.62 (6.82–15.46) *	65.6	8.33 ± 4.77	7.32 (5.04–10.34) *	<0.001
Intake of iron from various sources	Cereals (mg)	30.7	4.28 ± 3.19	3.35 (2.03–4.47) *	27.7	3.52 ± 2.19	3.04 (2.03–4.47) *	0.023
	Meat products (mg)	6.6	0.92 ± 2.45	0.00 (0.00–0.42) *	27.2	3.46 ± 3.62	2.17 (1.09–4.42) *	<0.001
	Vegetables (mg)	31.5	4.39 ± 3.41	3.23 (1.60–6.54) *	16.3	2.08 ± 1.84	1.60 (0.79–2.73) *	<0.001
	Nuts (mg)	10.7	1.50 ± 1.64	0.91 (0.18–2.26) *	7.9	1.00 ± 1.37	0.55 (0.00–1.45) *	0.004
	Fruit (mg)	7.3	1.02 ± 1.07	0.65 (0.37–1.13) *	5.5	0.70 ± 0.60	0.56 (0.28–0.92) *	0.014
	Cocoa products (mg)	3.7	0.51 ± 0.58	0.36 (0.14–0.75) *	4.1	0.53 ± 0.56	0.36 (0.18–0.67) *	0.783
	Eggs (mg)	3.6	0.50 ± 0.49	0.47 (0.16–0.63) *	4.1	0.53 ± 0.50	0.47 (0.31–0.63) *	0.281
	Potatoes (mg)	2.5	0.35 ± 0.46	0.21 (0.14–0.43) *	2.9	0.37 ± 0.35	0.29 (0.21–0.43) *	0.022
	Dairy products (mg)	2.2	0.31 ± 0.24	0.24 (0.13–0.47) *	2.1	0.27 ± 0.19	0.24 (0.15–0.35) *	0.469
	Fat (mg)	0.9	0.13 ± 0.13	0.09 (0.06–0.17) *	1.0	0.14 ± 0.13	0.11 (0.06–0.17) *	0.378
	Fish products (mg)	0.4	0.05 ± 0.09	0.00 (0.00–0.06) *	1.0	0.11 ± 0.16	0.06 (0.00–0.13) *	<0.001

* Nonparametric distribution (verified using Shapiro–Wilk test; *p* ≤ 0.05); ** compared using Mann–Whitney U test (due to nonparametric distribution).

**Table 7:** Comparison of the intake of various forms of iron along with that of iron intake from various sources in the national sample of Polish female adolescents, in the sub-groups of female respondents not applying and applying iron supplementation.

Intake of Iron		Female Respondents Not Applying Iron Supplementation (*n* = 1195)			Female Respondents Applying Iron Supplementation (*n* = 190)			*p*-Value **
		Intake (%)	Mean ± SD	Median (25th–75th)	Intake (%)	Mean ± SD	Median (25th–75th)	
Intake of various forms of iron	Total iron (mg)	100	12.56 ± 6.90	10.90 (7.77–15.52) *	100	14.32 ± 8.18	11.97 (8.86–16.96) *	0.006
	Heme-iron (mg)	13.2	1.66 ± 1.51	1.16 (0.70–2.11) *	12.8	1.84 ± 1.67	1.25 (0.75–2.25) *	0.176
	Non-heme iron (mg)	86.8	10.90 ± 5.89	9.50 (6.74–13.56) *	87.2	12.48 ± 7.22	10.69 (7.85–14.34) *	0.006
	Animal iron (mg)	33.0	4.15 ± 3.77	2.90 (1.76–5.28) *	32.1	4.60 ± 4.18	3.11 (1.88–5.61) *	0.176
	Plant iron (mg)	67.0	8.41 ± 4.88	7.36 (5.00–10.44) *	67.9	9.72 ± 6.35	8.17 (5.70–11.18) *	0.011
Intake of iron from various sources	Cereals (mg)	27.9	3.50 ± 2.20	3.01 (1.98–4.45) *	28.5	4.07 ± 2.76	3.35 (2.38–5.09) *	0.007
	Meat products (mg)	2.9	3.26 ± 3.56	2.00 (0.97–4.26) *	25.1	3.59 ± 3.89	2.17 (1.04–4.62) *	0.322
	Vegetables (mg)	17.4	2.19 ± 2.02	1.60 (0.79–2.89) *	17.0	2.43 ± 2.37	1.76 (0.79–3.18) *	0.300
	Nuts (mg)	8.0	1.00 ± 1.35	0.55 (0.00–1.45) *	8.6	1.22 ± 1.69	0.72 (0.18–1.45) *	0.111
	Fruit (mg)	5.5	0.69 ± 0.59	0.55 (0.28–0.92) *	6.4	0.91 ± 0.91	0.64 (0.37–1.02) *	0.010
	Cocoa products (mg)	4.1	0.52 ± 0.57	0.36 (0.18–0.63) *	4.1	0.58 ± 0.54	0.45 (0.21–0.79) *	0.050
	Eggs (mg)	4.1	0.52 ± 0.51	0.47 (0.31–0.63) *	4.0	0.57 ± 0.39	0.47 (0.31–0.75) *	0.001
	Potatoes (mg)	3.0	0.37 ± 0.36	0.29 (0.21–0.43) *	2.5	0.36 ± 0.35	0.29 (0.14–0.43) *	0.310
	Dairy products (mg)	2.1	0.27 ± 0.19	0.24 (0.14–0.34) *	2.1	0.31 ± 0.20	0.27 (0.18–0.39) *	0.003
	Fat (mg)	1.1	0.14 ± 0.14	0.11 (0.06–0.17) *	0.9	0.13 ± 0.12	0.11 (0.06–0.16) *	0.585
	Fish products (mg)	0.9	0.11 ± 0.16	0.06 (0.00–0.13) *	0.9	0.12 ± 0.17	0.06 (0.06–0.18) *	0.014

* Nonparametric distribution (verified using Shapiro–Wilk test; *p* ≤ 0.05); ** compared using Mann–Whitney U test (due to nonparametric distribution).

**Table 8:** Comparison of the intake of various forms of iron along with that of iron intake from various sources in the national sample of Polish female adolescents, in the sub-groups of female respondents from comprehensive and technical schools.

Intake of Iron		Female Respondents from Technical Schools (*n* = 893)			Female Respondents from Comprehensive Schools (*n* = 492)			*p*-Value **
		Intake (%)	Mean ± SD	Median (25th–75th)	Intake (%)	Mean ± SD	Median (25th–75th)	
Intake of various forms of iron	Total iron (mg)	100	12.42 ± 7.04	10.55 (7.47–15.52) *	100	13.41 ± 7.02	11.59 (8.95–16.15) *	0.001
	Heme-iron (mg)	14.7	1.82 ± 1.54	1.31 (0.77–2.37) *	14.6	1.96 ± 1.58	1.51 (0.84–2.54) *	0.043
	Non-heme iron (mg)	85.3	10.60 ± 5.97	9.02 (6.43–13.14) *	85.4	11.45 ± 5.94	10.03 (7.51–13.81) *	0.001
	Animal iron (mg)	36.6	4.55 ± 3.86	3.27 (1.94–5.92) *	36.5	4.90 ± 3.96	3.78 (2.09–6.36) *	0.043
	Plant iron (mg)	63.4	7.87 ± 4.85	6.83 (4.60–9.77) *	63.5	8.51 ± 4.87	7.46 (5.24–10.45) *	0.003
Intake of iron from various sources	Cereals (mg)	27.6	3.43 ± 2.47	2.94 (1.80–4.36) *	27.3	3.66 ± 2.31	3.21 (2.00–4.71) *	0.008
	Meat products (mg)	29.0	3.60 ± 3.59	2.27 (1.18–4.75) *	28.9	3.88 ± 3.74	2.75 (1.31–5.15) *	0.097
	Vegetables (mg)	16.1	2.00 ± 1.96	1.44 (0.63–2.73) *	16.1	2.16 ± 2.18	1.44 (0.63–2.57) *	0.433
	Nuts (mg)	6.6	0.82 ± 1.21	0.37 (0.00–1.08) *	6.5	0.87 ± 1.08	0.54 (0.00–1.45) *	0.164
	Fruit (mg)	4.8	0.60 ± 0.53	0.46 (0.28–0.74) *	5.2	0.69 ± 0.61	0.55 (0.28–0.83) *	0.004
	Cocoa products (mg)	4.0	0.49 ± 0.54	0.35 (0.18–0.63) *	4.0	0.54 ± 0.57	0.39 (0.18–0.67) *	0.098
	Eggs (mg)	4.5	0.56 ± 0.56	0.47 (0.31–0.79) *	4.6	0.62 ± 0.59	0.47 (0.31–0.79) *	0.014
	Potatoes (mg)	3.2	0.40 ± 0.40	0.29 (0.21–0.50) *	3.1	0.42 ± 0.44	0.29 (0.21–0.50) *	0.001
	Dairy products (mg)	2.2	0.27 ± 0.22	0.23 (0.13–0.35) *	1.3	0.29 ± 0.19	0.25 (0.16–0.37) *	0.015
	Fat (mg)	1.0	0.13 ± 0.14	0.09 (0.06–0.14) *	0.9	0.17 ± 0.21	0.11 (0.06–0.20) *	0.001
	Fish products (mg)	1.0	0.12 ± 0.19	0.06 (0.00–0.13) *	0.8	0.12 ± 0.17	0.06 (0.00–0.13) *	0.229

* Nonparametric distribution (verified using Shapiro–Wilk test; *p* ≤ 0.05); ** compared using Mann–Whitney U test (due to nonparametric distribution).

**Table 9:** Share of individuals characterized by adequate or inadequate iron intake.

Variable	Sub-Groups	<RDA *	>RDA *	*p*-Value **
Gender	Females	1038 (74.9%)	347 (25.1%)	<0.001
	Males	168 (16.4%)	857 (83.6%)	
Age	Minor females	715 (70.3%)	302 (29.7%)	<0.001
	Adult females	306 (83.2%)	62 (16.8%)	
Body mass index	Underweight females	145 (74.0%)	51 (26.0%)	0.312
	Proper body mass females	742 (73.9%)	262 (26.1%)	
	Overweight females	134 (72.4%)	61 (27.6%)	
Anemia history	Non-anemic females	855 (73.9%)	301 (26.1%)	0.231
	Anemic females	160 (67.7%)	69 (30.3%)	
Following vegetarian diet	Non-vegetarian females	999 (76.8%)	301 (23.2%)	0.236
	Vegetarian females	60 (70.6%)	25 (29.4%)	
Applying iron supplementation	Females not applying iron supplementation	888 (74.3%)	307 (25.7%)	0.244
	Females applying iron supplementation	133 (70.0%)	57 (30.0%)	
Type of school	Females from comprehensive schools	369 (75.0%)	123 (25.0%)	0.281
	Females from technical schools	694 (77.7%)	199 (22.3%)	

* RDA (recommended dietary allowance; for females aged 15–18—15 mg, females aged 19–20—18 mg, males aged 15–18—11 mg, males aged 19–20—8 mg [21]), ** compared using chi^2^ test.

## Supplementary Materials

The following are available online at https://www.mdpi.com/2072-6643/11/5/1049/s1, Material S1: Detailed methodology of the recruitment procedure; Table S1: Comparison of the intake of various forms of iron along with that of iron intake from various sources in the national sample of Polish male adolescents, in the sub-groups of minor and adult male respondents; Table S2: Comparison of the intake of various forms of iron along with that of iron intake from various sources in the national sample of Polish male adolescents, in the sub-groups of underweight, proper body mass, and overweight male respondents; Table S3: Comparison of the intake of various forms of iron along with that of iron intake from various sources in the national sample of Polish male adolescents, in the sub-groups of non-anemic and anemic history male respondents; Table S4: Comparison of the intake of various forms of iron along with that of iron intake from various sources in the national sample of Polish male adolescents, in the sub-groups of non-vegetarian and vegetarian male respondents; Table S5: Comparison of the intake of various forms of iron along with that of iron intake from various sources in the national sample of Polish male adolescents, in the sub-groups of male respondents applying no iron supplementation and male respondents applying iron supplementation; Table S6: Comparison of the intake of various forms of iron along with that of iron intake from various sources in the national sample of Polish male adolescents, in the sub-groups of male respondents from comprehensive and technical schools; Table S7: Share of male individuals characterized by adequate or inadequate iron intake.
