# Supplementary material for: Analysis of Heme and Non-Heme Iron Intake and Iron Dietary Sources in Adolescent Menstruating Females in a National Polish Sample
Source: Nutrients. 2019 May 10;11(5):1049. doi: 10.3390/nu11051049 (PMC6567869; doi:10.3390/nu11051049)
Supplement: Supplementary file 1 [file nutrients-11-01049-s001.pdf]

nutrients

MDPI

Article

Analysis of Heme and Non-Heme Iron Intake and Iron Dietary Sources in Adolescent Menstruating Females in a National Polish Sample

Dominika Skolmowska and Dominika Głąbska

Supplementary Material S1. Detailed methodology of the recruitment procedure.

The main phase included:

- Random selection of 5 counties out of each of the 16 voivodeships (80 counties were randomly selected out of 380 counties in Poland);
- Random selection of 5 secondary schools out of each of the 80 counties (400 secondary schools were randomly selected out of 14,009 secondary schools in Poland).

After the main phase, the principal of each randomly selected secondary school received a letter with invitation to participate and an e-mail with all necessary additional information, such as detailed aims of the study and its course. After two weeks, they received additional reminders to determine whether their schools (i.e., students from their schools) were interested to participate in the study. After confirmation, they received a written informed consent form, which was to be gathered from students and their parents/legal guardians for minors. Then, they received a link to the electronic version of the questionnaire with an access code that was generated for those students who provided written informed consent.

After one month of data gathering, the distribution of participants was analyzed and evaluated and the subsidiary phase of the sampling procedure was conducted. The subsidiary phase included:

- For voivodeships with less than 50 respondents gathered (9 voivodeships): Random selection of 10 counties out of each of the 9 voivodeships (90 counties were randomly selected);
- For voivodeships with more than 50 respondents gathered (7 voivodeships): Random selection of 5 counties out of each of the 7 voivodeships (35 counties were randomly selected);
- Random selection of 5 secondary schools out of each of the 125 counties (625 secondary schools were randomly selected).

After the subsidiary phase, similar to the main phase, the principal of each randomly selected secondary school received a letter with invitation to participate and an e-mail with all necessary additional information. After two weeks, a reminder was also issued. If the school's participations for the study is confirmed, they received a written informed consent form and a link to an electronic version of the questionnaire. Moreover, an access code was generated for the school's students. After this stage, data were gathered for six weeks.

After the whole procedure, 2514 participants completed the questionnaire with no missing data, which were assessed after considering previously established inclusion and exclusion criteria. The necessary information was gathered using written forms provided by students and their parents/legal guardians for minors, as well as using a brief online questionnaire (general characteristics such as sociodemographic data).

Supplementary Table S1. Comparison of the intake of various forms of iron along with that of iron intake from various sources in the national sample of Polish male adolescents, in the sub-groups of minor and adult male respondents.

Intake of iron		Minor male respondents (n = 722)			Adult male respondents (n = 303)			p-Value **
		Intake (%)	Mean ± SD	Median (25th–75th)	Intake (%)	Mean ± SD	Median (25th–75th)	
Intake of various forms of iron	Total iron (mg)	100	17.91 ± 9.28	15.59 (11.20–23.00) *	100	17.06 ± 9.01	15.26 (10.19–21.89) *	0.180
	Heme-iron (mg)	17.5	3.14 ± 2.23	2.54 (1.54–4.19) *	16.2	2.76 ± 1.97	2.17 (1.40–3.85) *	0.014
	Non-heme iron (mg)	82.5	14.77 ± 7.65	12.85 (9.24–4.06) *	83.8	14.30 ± 7.65	12.65 (8.61–18.38) *	0.273
	Animal iron (mg)	43.9	7.86 ± 5.59	6.35 (3.86–10.46) *	40.55	6.91 ± 4.93	5.43 (3.49–9.63) *	0.014
	Plant iron (mg)	56.1	10.06 ± 5.99	8.79 (5.74–12.66) *	59.5	10.15 ± 6.26	8.34 (5.23–13.58) *	0.850
Intake of iron from various sources	Cereals (mg)	24.9	4.46 ± 3.09	3.85 (2.29–5.76) *	27.0	4.61 ± 3.41	3.78 (2.10–6.28) *	0.908
	Meat products (mg)	36.1	6.47 ± 5.23	5.15 (2.69–8.74) *	31.5	5.37 ± 4.40	4.06 (2.05–7.50) *	0.002
	Vegetables (mg)	13.7	2.45 ± 2.39	1.91 (0.79–3.39) *	14.9	2.54 ± 2.38	1.76 (1.13–3.23) *	0.518
	Nuts (mg)	5.9	1.06 ± 1.54	0.72 (0.04–1.45) *	6.1	1.03 ± 1.60	0.72 (0.00–1.36) *	0.829
	Fruit (mg)	4.0	0.72 ± 0.68	0.55 (0.28–0.92) *	3.9	0.67 ± 0.70	0.46 (0.28–0.83) *	0.010
	Cocoa products (mg)	3.6	0.64 ± 0.68	0.45 (0.21–0.82) *	3.3	0.57 ± 0.72	0.33 (0.15–0.75) *	0.001
	Eggs (mg)	4.7	0.84 ± 0.85	0.63 (0.31–0.94) *	5.9	1.01 ± 1.08	0.63 (0.31–1.26) *	0.032
	Potatoes (mg)	3.1	0.55 ± 0.62	0.36 (0.21–0.64) *	3.2	0.55 ± 0.59	0.36 (0.21–0.68) *	0.901
	Dairy products (mg)	2.0	0.35 ± 0.26	0.28 (0.18–0.44) *	2.1	0.35 ± 0.30	0.27 (0.16–0.47) *	0.247
	Fat (mg)	0.9	0.17 ± 0.22	0.11 (0.06–0.20) *	1.0	0.18 ± 0.23	0.11 (0.06–0.20) *	0.829
	Fish products (mg)	1.2	0.20 ± 0.28	0.13 (0.06–0.22) *	1.0	0.18 ± 0.26	0.06 (0.00–0.22) *	0.095

* Nonparametric distribution (verified using Shapiro–Wilk test; $p \leq 0.05$), ** compared using Mann–Whitney U test (due to nonparametric distribution).

Supplementary Table S2. Comparison of the intake of various forms of iron along with that of iron intake from various sources in the national sample of Polish male adolescents, in the sub-groups of underweight, proper body mass, and overweight male respondents.

Intake of iron		Underweight male respondents (n = 54)			Proper body mass male respondents (n = 708)			Overweight male respondents (n = 263)			p-Value **
		Intake (%)	Mean ± SD	Median (25th–75th)	Intake (%)	Mean ± SD	Median (25th–75th)	Intake (%)	Mean ± SD	Median (25th–75th)	
Intake of various forms of iron	Total iron (mg)	100	17.16 ± 9.65	14.46 (10.70–21.27) *	100	17.82 ± 9.20	15.74 (11.05–22.77) *	100	17.33 ± 9.15	15.29 (10.83–22.50) *	0.551
	Heme-iron (mg)	16.0	2.74 ± 1.96	2.59 (1.16–3.29) *	16.9	3.02 ± 2.09	2.47 (1.51–4.17) *	18.0	3.12 ± 2.40	2.32 (1.49–3.98) *	0.656
	Non-heme iron (mg)	84.0	14.42 ± 8.19	12.61 (8.14–17.36) *	83.1	14.80 ± 7.71	12.91 (9.20–18.87) *	82.0	14.21 ± 7.38	12.61 (8.76–18.31) *	0.571
	Animal iron (mg)	39.9	6.85 ± 4.91	6.48 (2.90–8.23) *	42.4	7.55 ± 5.23	6.17 (3.77–10.43) *	45.0	7.80 ± 5.99	5.80 (3.71–9.95) *	0.656
	Plant iron (mg)	60.1	10.31 ± 6.51	9.15 (5.71–12.30) *	57.6	10.27 ± 6.17	8.80 (5.56–13.25) *	55.0	9.54 ± 5.66	8.21 (5.69–11.60) *	0.339
Intake of iron from various sources	Cereals (mg)	28.3	4.85 ± 3.47	4.41 (1.98–6.90) *	36.2	4.64 ± 3.28	3.86 (2.34–6.04) *	23.5	4.08 ± 2.82	3.46 (2.12–5.31) *	0.053
	Meat products (mg)	33.1	5.68 ± 4.47	5.33 (2.41–7.17) *	34.1	6.07 ± 4.84	4.75 (2.49–8.42) *	37.2	6.44 ± 5.58	4.58 (2.65–8.23) *	0.896
	Vegetables (mg)	14.1	2.42 ± 2.91	1.59 (0.51–3.23) *	13.8	2.46 ± 2.38	1.76 (0.94–3.23) *	14.7	2.54 ± 2.27	2.07 (1.13–3.39) *	0.367
	Nuts (mg)	4.4	0.76 ± 0.99	0.37 (0.00–1.22) *	5.9	1.06 ± 1.64	0.72 (0.00–1.45) *	6.3	1.09 ± 1.40	0.72 (0.18–1.45) *	0.061
	Fruit (mg)	4.4	0.75 ± 0.76	0.42 (0.28–0.92) *	3.9	0.69 ± 0.68	0.46 (0.28–0.92) *	4.1	0.72 ± 0.68	0.55 (0.28–0.92) *	0.877
	Cocoa products (mg)	3.8	0.66 ± 0.74	0.53 (0.18–0.89) *AB	3.7	0.66 ± 0.75	0.45 (0.21–0.88) *A	2.8	0.49 ± 0.46	0.36 (0.21–0.63) *B	0.020
	Eggs (mg)	3.9	0.67 ± 0.83	0.47 (0.31–0.79) *A	5.2	0.92 ± 0.94	0.63 (0.31–1.10) *B	4.8	0.84 ± 0.91	0.63 (0.31–0.94) *AB	0.007
	Potatoes (mg)	3.9	0.66 ± 0.77	0.36 (0.21–0.70) *	3.2	0.57 ± 0.65	0.36 (0.21–0.63) *	2.7	0.47 ± 0.42	0.36 (0.21–0.57) *	0.307
	Dairy products (mg)	2.2	0.38 ± 0.27	0.29 (0.21–0.47) *	2.0	0.3 6 ± 0.28	0.28 (0.18–0.44) *	1.9	0.33 ± 0.27	0.26 (0.17–0.41) *	0.210
	Fat (mg)	1.2	0.20 ± 0.24	0.14 (0.06–0.22) *	1.1	0.18 ± 0.23	0.11 (0.06–0.20) *	0.9	0.15 ± 0.19	0.10 (0.06–0.17) *	0.283
	Fish products (mg)	0.7	0.13 ± 0.20	0.06 (0.00–0.19) *A	1.0	0.20 ± 0.28	0.13 (0.06–0.22) *B	1.1	0.19 ± 0.25	0.13 (0.00–0.22) *AB	0.030

* Nonparametric distribution (verified using Shapiro–Wilk test; $p \leq 0.05$), ** Compared using Kruskal–Wallis analysis of variance (ANOVA) (due to nonparametric distribution), values with different letters (A, B) differ in rows.

Supplementary Table S3. Comparison of the intake of various forms of iron along with that of iron intake from various sources in the national sample of Polish male adolescents, in the sub-groups of anemic and non-anemic history male respondents.

Intake of iron		Anemic history male respondents (*n* = 41)			Non-anemic history male respondents (*n* = 984)			*p*-Value **
		Intake (%)	Mean ± SD	Median (25th–75th)	Intake (%)	Mean ± SD	Median (25th–75th)	
Intake of various forms of iron	Total iron (mg)	100	17.82 ± 9.29	15.05 (10.97–22.52) *	100	17.66 ± 9.21	15.59 (11.00–22.50) *	0.847
	Heme-iron (mg)	16.8	3.00 ± 2.30	2.42 (1.48–4.05) *	17.2	3.03 ± 2.16	2.44 (1.48–4.03) *	0.705
	Non-heme iron (mg)	83.2	14.82 ± 7.58	13.72 (9.05–18.71) *	82.8	14.62 ± 7.66	12.77 (9.08–18.72) *	0.951
	Animal iron (mg)	42.1	7.51 ± 5.74	6.06 (3.69–10.12) *	42.9	7.58 ± 5.40	6.10 (3.71–10.09) *	0.705
	Plant iron (mg)	57.9	10.31 ± 5.83	9.15 (5.67–12.99) *	57.1	10.08 ± 6.08	8.67 (5.67–12.93) *	0.823
Intake of iron from various sources	Cereals (mg)	24.8	4.42 ± 3.07	3.89 (2.25–5.97) *	25.5	4.51 ± 3.19	3.80 (2.25–6.01) *	0.884
	Meat products (mg)	34.2	6.10 ± 5.38	4.55 (2.53–8.21) *	34.8	6.15 ± 5.01	4.75 (2.56–8.10) *	0.703
	Vegetables (mg)	16.2	2.89 ± 2.65	2.26 (0.96–3.39) *	13.9	2.46 ± 2.37	1.76 (0.94–3.39) *	0.356
	Nuts (mg)	4.7	0.83 ± 1.07	0.45 (0.00–1.45) *	6.0	1.06 ± 1.57	0.72 (0.00–1.45) *	0.209
	Fruit (mg)	4.3	0.76 ± 1.00	0.56 (0.28–0.92) *	4.0	0.70 ± 0.67	0.46 (0.28–0.92) *	0.741
	Cocoa products (mg)	3.6	0.64 ± 0.72	0.45 (0.21–0.78) *	3.5	0.62 ± 0.69	0.42 (0.21–0.76) *	0.830
	Eggs (mg)	4.9	0.87 ± 0.87	0.55 (0.31–1.10) *	5.0	0.89 ± 0.93	0.63 (0.31–1.10) *	0.580
	Potatoes (mg)	3.3	0.59 ± 0.80	0.36 (0.21–0.64) *	3.1	0.55 ± 0.60	0.36 (0.21–0.64) *	0.463
	Dairy products (mg)	1.7	0.30 ± 0.22	0.25 (0.18–0.44) *	2.0	0.35 ± 0.28	0.28 (0.18–0.44) *	0.162
	Fat (mg)	0.1	0.19 ± 0.20	0.11 (0.06–0.20) *	1.0	0.17 ± 0.22	0.11 (0.06–0.20) *	0.216
	Fish products (mg)	1.3	0.24 ± 0.30	0.13 (0.00–0.22) *	1.1	0.19 ± 0.27	0.13 (0.00–0.22) *	0.533

* Nonparametric distribution (verified using Shapiro–Wilk test; $p \leq 0.05$), ** Compared using Mann–Whitney U test (due to nonparametric distribution).

Supplementary Table S4. Comparison of the intake of various forms of iron along with that of iron intake from various sources in the national sample of Polish male adolescents, in the sub-groups of vegetarian and non-vegetarian male respondents.

Intake of iron		Vegetarian male respondents (*n* = 26)			Non-vegetarian male respondents (*n* = 999)			*p*-Value **
		Intake (%)	Mean ± SD	Median (25th–75th)	Intake (%)	Mean ± SD	Median (25th–75th)	
Intake of various forms of iron	Total iron (mg)	100	14.68 ± 10.18	13.23 (4.22–22.80) *	100	17.74 ± 9.17	15.62 (11.01–22.53) *	0.051
	Heme-iron (mg)	13.0	1.91 ± 2.19	1.03 (0.65–2.43) *	17.2	3.06 ± 2.16	2.47 (1.52–4.07) *	0.001
	Non-heme iron (mg)	87.0	12.77 ± 8.61	12.17 (3.63–22.18) *	82.8	14.68 ± 7.62	12.80 (9.15–18.69) *	0.176
	Animal iron (mg)	32.4	4.76 ± 5.47	2.57 (1.63–6.08) *	43.1	7.65 ± 5.40	6.17 (3.80–10.17) *	0.001
	Plant iron (mg)	67.6	9.92 ± 6.89	10.27 (2.80–12.61) *	56.9	10.09 ± 6.05	8.67 (5.68–12.99) *	0.898
Intake of iron from various sources	Cereals (mg)	25.5	3.84 ± 3.08	3.59 (1.22–5.21) *	25.5	4.52 ± 3.19	3.80 (2.27–6.02) *	0.226
	Meat products (mg)	35.0	3.60 ± 4.93	2.08 (0.60–3.72) *	35.0	6.21 ± 5.01	4.84 (2.60–8.27) *	0.001
	Vegetables (mg)	13.9	2.88 ± 2.63	2.26 (1.01–4.47) *	13.9	2.47 ± 2.38	1.76 (0.96-3.31) *	0.461
	Nuts (mg)	5.9	1.44 ± 2.49	0.73 (0.00–1.95) *	5.9	1.04 ± 1.52	0.72 (0.00–1.45) *	0.771
	Fruit (mg)	4.0	0.71 ± 1.01	0.37 (0.21–0.85) *	4.0	0.70 ± 0.68	0.46 (0.28–0.92) *	0.199
	Cocoa products (mg)	3.5	0.53 ± 0.44	0.42 (0.28–0.60) *	3.5	0.62 ± 0.70	0.42 (0.21–0.79) *	0.987
	Eggs (mg)	5,0	0.68 ± 0.74	0.47 (0.20–0.79) *	5.0	0.89 ± 0.93	0.63 (0.31–1.10) *	0.091
	Potatoes (mg)	3.1	0.39 ± 0.44	0.25 (0.14–0.46) *	3.1	0.56 ± 0.61	0.36 (0.21–0.64) *	0.026
	Dairy products (mg)	2.0	0.30 ± 0.27	0.23 (0.16–0.31) *	2.0	0.35 ± 0.28	0.28 (0.18–0.44) *	0.158
	Fat (mg)	0.8	0.12 ± 0.11	0.09 (0.04–0.14) *	1.0	0.18 ± 0.22	0.11 (0.06–0.20) *	0.163
	Fish products (mg)	1.2	0.17 ± 0.40	0.06 (0.00–0.18) *	1.1	0.20 ± 0.27	0.13 (0.04–0.22) *	0.058

* Nonparametric distribution (verified using Shapiro–Wilk test; $p \leq 0.05$), ** Compared using Mann–Whitney U test (due to nonparametric distribution).

Supplementary Table S5. Comparison of the intake of various forms of iron along with that of iron intake from various sources in the national sample of Polish male adolescents, in the sub-groups of male respondents not applying and applying iron supplementation.

Intake of iron		Male respondents not applying iron supplementation (n = 902)			Male respondents applying iron supplementation (n = 123)			p-Value **
		Intake (%)	Mean ± SD	Median (25th–75th)	Intake (%)	Mean ± SD	Median (25th–75th)	
Intake of various forms of iron	Total iron (mg)	100	17.25 ± 7.34	15.13 (10.67–21.97) *	100	20.65 ± 9.79	19.70 (13.28–27.32) *	0.001
	Heme-iron (mg)	17.2	2.96 ± 1.55	2.36 (1.43–3.93) *	17.0	3.52 ± 2.14	3.26 (1.96–4.52) *	0.001
	Non-heme iron (mg)	82.8	14.29 ± 6.39	12.52 (8.82–18.03) *	83.0	17.13 ± 8.34	15.67 (11.34–22.79) *	0.001
	Animal iron (mg)	43.0	7.41 ± 3.97	5.89 (3.58–9.83) *	42.7	8.81 ± 5.36	8.15 (4.91–11.30) *	0.001
	Plant iron (mg)	57.0	9.85 ± 5.45	8.54 (5.57–12.62) *	57.3	11.84 ± 6.91	10.30 (6.55–15.13) *	0.002
Intake of iron from various sources	Cereals (mg)	25.6	4.41 ± 2.41	3.76 (2.20–5.94) *	2.2	5.21 ± 4.12	4.43 (2.83–6.47) *	0.046
	Meat products (mg)	35.0	6.05 ± 3.63	4.58 (2.35–8.01) *	33.2	6.85 ± 4.96	5.71 (3.33–9.32) *	0.018
	Vegetables (mg)	13.9	2.40 ± 2.11	1.60 (0.94–3.23) *	14.7	3.05 ± 2.61	2.41 (1.26–3.70) *	0.002
	Nuts (mg)	5.9	1.02 ± 1.60	0.72 (0.00–1.45) *	6.2	1.29 ± 1.74	0.72 (0.37–1.53) *	0.018
	Fruit (mg)	3.8	0.66 ± 0.73	0.46 (0.28–0.83) *	4.8	1.00 ± 1.00	0.65 (0.46–1.20) *	0.001
	Cocoa products (mg)	3.5	0.61 ± 0.59	0.42 (0.21–0.76) *	3.2	0.65 ± 0.74	0.45 (0.21–0.83) *	0.491
	Eggs (mg)	4.8	0.84 ± 0.53	0.63 (0.31–0.94) *	6.2	1.27 ± 1.29	0.79 (0.47–1.57) *	0.001
	Potatoes (mg)	3.3	0.56 ± 0.38	0.36 (0.21–0.64) *	2.3	0.47 ± 0.37	0.36 (0.21–0.50) *	0.077
	Dairy products (mg)	2.1	0.34 ± 0.20	0.28 (0.18–0.43) *	2.2	0.45 ± 0.36	0.32 (0.20–0.58) *	0.001
	Fat (mg)	1.0	0.17 ± 0.15	0.11 (0.06–0.20) *	0.9	0.18 ± 0.29	0.09 (0.06–0.20) *	0.102
	Fish products (mg)	1.1	0.19 ± 0.17	0.13 (0.01–0.22) *	1.1	0.23 ± 0.29	0.15 (0.03–0.32) *	0.042

* Nonparametric distribution (verified using Shapiro–Wilk test; $p \leq 0.05$), ** Compared using Mann–Whitney U test (due to nonparametric distribution).

Supplementary Table S6. Comparison of the intake of various forms of iron along with that of iron intake from various sources in the national sample of Polish male adolescents, in the sub-groups of male respondents from technical and comprehensive schools.

Intake of iron		Male respondents from technical schools (*n* =234)			Male respondents from comprehensive schools (*n* = 791)			*p*-Value **
		Intake (%)	Mean ± SD	Median (25th–75th)	Intake (%)	Mean ± SD	Median (25th–75th)	
Intake of various forms of iron	Total iron (mg)	100	17.56 ± 9.16	15.51 (10.87–22.47) *	100	13.63 ± 7.54	12.19 (11.26–23.39) *	0.721
	Heme-iron (mg)	15.7	2.76 ± 2.25	2.07 (1.10–3.81) *	15.1	2.06 ± 1.80	1.50 (1.21–3.88) *	0.671
	Non-heme iron (mg)	84.3	14.81 ± 7.55	12.99 (9.30–19.10) *	84.9	11.59 ± 6.29	10.79 (9.68–18.82) *	0.718
	Animal iron (mg)	39.2	6.89 ± 5.62	5.19 (2.74–9.52) *	37.9	5.16 ± 4.49	3.75 (3.02–9.70) *	0.671
	Plant iron (mg)	60.8	10.67 ± 5.98	9.36 (6.38–13.51) *	62.1	8.55 ± 5.08	7.69 (6.68–13.04) *	0.881
Intake of iron from various sources	Cereals (mg)	26.2	4.60 ± 2.96	3.89 (2.83–6.47) *	25.3	3.67 ± 2.68	3.19 (2.39–5.61) *	0.310
	Meat products (mg)	31.7	5.56 ± 5.18	3.93 (3.33–9.32) *	31.8	5.70 ± 5.27	3.96 (1.76–8.03) *	0.708
	Vegetables (mg)	15.2	2.67 ± 2.33	2.10 (1.26–3.70) *	15.6	2.23 ± 2.21	1.68 (1.13–3.54) *	0.410
	Nuts (mg)	7.4	1.29 ± 1.62	0.73 (0.37–1.53) *	7.6	1.00 ± 1.50	0.72 (0.18–1.81) *	0.525
	Fruit (mg)	4.7	0.82 ± 0.74	0.65 (0.46–1.20) *	4.5	0.67 ± 0.70	0.46 (0.37–0.93) *	0.394
	Cocoa products (mg)	3.6	0.63 ± 0.67	0.42 (0.21–0.83) *	3.8	0.51 ± 0.58	0.36 (0.21–0.87) *	0.848
	Eggs (mg)	4.6	0.81 ± 0.90	0.63 (0.47–1.57) *	4.5	0.62 ± 0.69	0.47 (0.31–0.94) *	0.842
	Potatoes (mg)	2.8	0.50 ± 0.54	0.36 (0.21–0.50) *	3.0	0.42 ± 0.49	0.29 (0.21–0.57) *	0.749
	Dairy products (mg)	2.0	0.34 ± 0.26	0.28 (0.20–0.50) *	1.9	0.29 ± 0.23	0.24 (0.18–0.44) *	0.771
	Fat (mg)	1.0	0.17 ± 0.19	0.11 (0.06–0.20) *	1.0	0.13 ± 0.17	0.09 (0.06–0.20) *	0.552
	Fish products (mg)	1.0	0.17 ± 0.25	0.13 (0.03–0.32) *	1.0	0.14 ± 0.23	0.06 (0.06–0.22) *	0.404

* Nonparametric distribution (verified using Shapiro–Wilk test; $p \leq 0.05$), ** Compared using Mann–Whitney U test (due to nonparametric distribution).

Supplementary Table S7. Share of individuals characterized by adequate or inadequate iron intake.

Variable	Sub-groups	<RDA *	>RDA *	*p*-Value **
Age	Minor males	133 (18.4%)	589 (81.6%)	0.760
	Adult males	59 (19.5%)	244 (80.5%)	
Body mass index	Underweight males	12 (22.2%)	42 (77.8%)	0.309
	Proper body mass males	128 (18.1%)	580 (81.9%)	
	Overweight males	44 (16.7%)	219 (83.3%)	
Anemia history	Non-anemic males	164 (16.7%)	820 (83.3%)	0.588
	Anemic males	5 (12.2%)	36 (87.8%)	
Following vegetarian diet	Non-vegetarian males	160 (16.0%)	839 (84.0%)	1.000
	Vegetarian males	4 (15.4%)	22 (84.6%)	
Applying iron supplementation	Males not applying iron supplementation	152 (16.9%)	750 (83.1%)	0.874
	Males applying iron supplementation	22 (17.9%)	101 (82.1%)	
Type of school	Males from comprehensive schools	34 (14.5%)	200 (85.5%)	0.739
	Males from technical schools	106 (13.4%)	685 (86.6%)	

* RDA (recommended dietary allowance; for males aged 15–18—11 mg, males aged 19–20—8 mg [21]), ** Compared using chi^2 test.
